# Dr. David Skae’s Definition of Insanity

**Published:** 1861-08

**Authors:** 


### DR. DAVID SKAE'S DEFINITION OF INSANITY.

I had arrived at the conclusion that it was a disease of the brain affecting the mind. I have to complete this definition by saying how it affects the mind. My reply is, that emotions and passions are caused by the *disease*, and not by the motives ordinarily calling into action these emotions—that is *moral* insanity—and that in another class of cases ideas are believed in which have no evidence of their truth; they are neither founded on fact, observation, nor memory, and are such as no sane man would entertain as matters of testimony or observation; they are, in fact, morbid fancies and beliefs—ideas caused and believed in by disease.

To reduce my definition to a brief compass, I would say that *insanity is an* (*apyretic*) *affection of the brain in which emotions*, *passions*, *or desires are excited by* DESEASE (*not by motives*,) *or in which* CONCEPTIONS *are mistaken for acts of* PERCEPTION OF MEMORY.

This definition appears to me to comprise everything. The first part of it defines *moral insanity*, in which the propensities, emotions, and desires alone are *morbidly* excited; and the second part of it defines *intellectual insanity*, in which there are actual delusions or hallucinations, so long considered the essential feature of madness. If I would add anything to this definition, it would be the *loss of self-control* or *self-direction*, which appears to me to be the peculiar characteristic of all forms of insanity,—that loss of self-control over the actions, which permits them to be restless, violent, or extravagant; a loss of control over the passions, which permits them to overrule the judgment and the conscience, and ends in acts of vice, debasement or violence; a loss of control over the succession of the thoughts, which permits them to be incessant, rapid, and incoherent; a loss of control over the ideas, which precludes the insane from the exercise of comparison and judgment, and leaves them (as D. Stewart remarked), like persons in dreams, to mistake the objects of reverie or imagination for realities. In fact, I know of no designation for insanity which more briefly and correctly distinguishes it than the old Scotch one, namely, a man who has *lost his judgment*.—*Edin. Med. Journal*, *April*, 1861.

